# Azacytidine and Venetoclax in Relapsed and Refractory Patients With Angioimmunoblastic T-cell Lymphoma

**DOI:** 10.1097/HS9.0000000000000675

**Published:** 2022-02-01

**Authors:** Kamel Laribi, Alix Baugier de Materre, Yamina Touileb, Charles Boursot, Jeremy Sandrini, Doriane Cavalieri, Cédric Pastoret, Laurence de Leval, Olivier Tournilhac

**Affiliations:** 1 Department of Haematology, Centre Hospitalier Le Mans, France; 2 Geriatric Department, Assistance Publique-Hôpitaux de Paris, Hôpital Broca, Paris, France; 3 Department of Nuclear Medicine, Centre Hospitalier Le Mans, France; 4 Department of Anatomopathology, Centre Hospitalier Le Mans, France; 5 Haematology and Cell Therapy Department, Hôpital Estaing, CHU de Clermont-Ferrand, France; 6 Clermont Auvergne University, Clermont-Ferrand, France; 7 Laboratoire d'Hématologie, Centre Hospitalier Universitaire de Rennes, France; 8 Department of Laboratory Medicine and Pathology, Institute of Pathology, Lausanne University Hospital and Lausanne University, Switzerland

Angioimmunoblastic T-cell lymphoma (AITL) is a nodal T-cell lymphoma with a T-follicular helper (TFH) phenotype and aggressive clinical behavior. Molecular studies have shown recurrent mutations in *TET2*, *DNMT3A*, *RHOA*, and *IDH1/2* in a significant proportion of cases.^1,2^ Treatment in the front-line setting is most frequently anthracycline-based regimen, which is associated with a high failure rate and frequent relapses. The prognosis for patients with relapsed/refractory (R/R) disease is poor with a median overall survival (OS) of 6 months.^3^ Hypomethylating agents (HMA) are the main treatment of high-risk myelodysplastic syndrome (MDS) and acute myeloid leukemia (AML) in elderly patients, and the response rate to HMAs were correlated with *TET2*, *IDH1/2*, and *DNMT3A* mutations.^4^

Activity of HMAs against TFH-derived peripheral T-cell lymphoma (PTCL) was shown in previous case reports.^5^ The Lymphoma Study Association (LYSA) group reported a series of 12 patients with AITL treated with 5-azacytidine. Concomitant myeloid neoplasm (MDS/CMML) was present in 5 patients. The overall response (OR) and complete response (CR) rates were 75% and 50%, respectively. After a median follow-up of 27 months, the median progression-free survival (PFS) and OS were 15 months and 21 months, respectively.^6^

Overexpression of the antiapoptotic protein B-cell lymphoma 2(Bcl-2) was reported in AITL patients (43%–86%) and was strongly associated with advanced stage and higher international prognostic indices (IPI).^7,8^

Venetoclax is a selective and orally bioavailable small-molecule inhibitor of BCL-2, US-FDA approved alone or in combination in CLL and AML.

Previous reports have shown that patient-derived cutaneous T-cell lymphoma (CTCL) cells exhibit a variable sensitivity to venetoclax correlated with baseline Bcl-2.^9^

King et al treated one R/R mycosis fungoides-CTCL patient with venetoclax monotherapy. The patient achieved PR. *In vitro* viability assays followed after 6 months of treatment showed no significant change in drug sensitivity, consistent with the absence of development of resistance to venetoclax.^10^

Here, we report the efficacy and safety of 5-azacytidine administered at 75 mg/m^2^ daily, subcutaneously, for 7 consecutive days, every 28 days, plus venetoclax administered at 400 mg daily, after dose escalation (100 mg at day 1, 200 mg at day 2, then 400 mg daily), until progression or unacceptable toxicity, in 5 patients with R/R AITL, enrolled in 2 centers in France, between April 2020 and February 2021.

AITL diagnoses were all confirmed by 2 expert pathologists in the framework of the national program “Lymphopath,” based on the criteria of the World Health Organization 2016 classification. By immunohistochemistry, the lymphoma cells had a CD10+PD1+ BCL6+ CXCL13± phenotype. Expression of BCL-2 was moderate in PD1– atypical cells in 3 cases and low in 2 cases compared with the level of expression of small reactive lymphoid cells (Figure 1).

**Figure 1.**
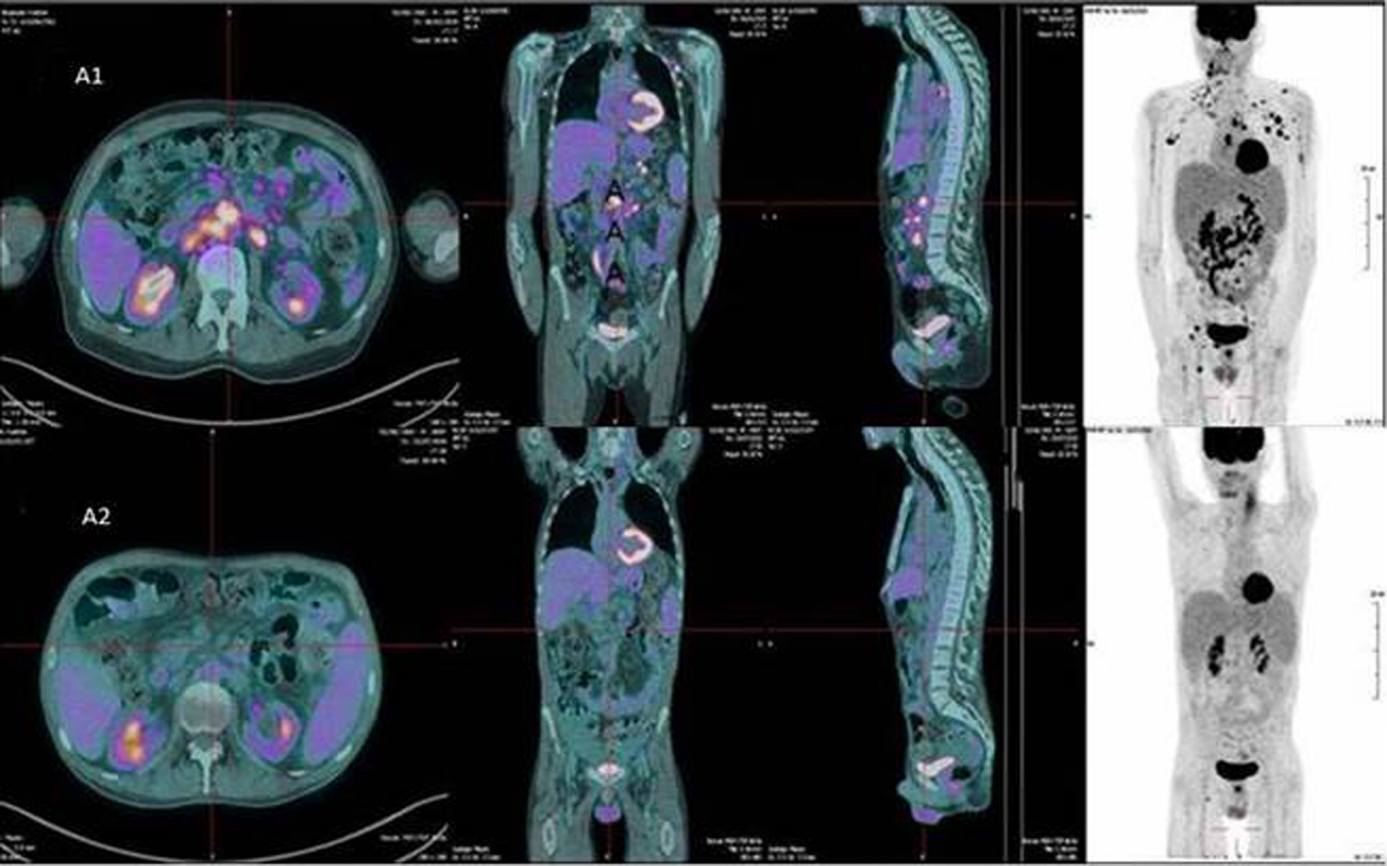
**FDG/PET imaging in patient 1.** (A) FDG/PET imaging showing complete metabolic response after 3 cycles of AZA+VEN with disappearance of lymph nod and bone lesions. (B) FDG/PET imaging before AZA+VEN initiation showing cervical, axillary, mediastinal, mesenteric, retroperintoneal, and bone involvement. AZA+VEN = azacytidine and venetoclax; FDG/PET = fluorodeoxyglucose positron emission tomography.

Molecular analysis was performed on lymph node biopsies collected at initial diagnosis. DNA was extracted from frozen or FFPE lymph node biopsies with a Maxwell Rapid Sample Concentrator (Promega, Madison, WI). We sequenced a panel of 16 genes dedicated to T-cell lymphomas (*CARD11*, *CD28*, *DNMT3A*, *IDH1*, *IDH2*, *JAK3*, *KRAS*, *NRAS*, *PLCG1*, *RHOA*, *SETD2*, *STAT3*, *STAT5B*, *TET2*, *TNFAIP3*, and *TP53*) from 100 ng of DNA. Libraries were generated in duplicate using an amplicon-based strategy with Advanta NGS Library Prep reagents on an Access ArrayTM 48.48 Integrated Fluidic Circuit (Fluidigm, San Francisco, CA) and sequenced on a NextSeq550 platform (Illumina, San Diego, CA) with a median coverage >1000×. Data were analyzed with a custom bioinformatic pipeline. Exonic nonsynonymous mutations with a variant allelic frequency >1% and at least 20 mutated reads were reported.

Tumor responses included physical examination, chest and abdominal CT, and PET-CT and responses were assessed after 3 and 6 cycles of therapy by the attending physician following the 1999 Cheson criteria.^11^ The study was approved by a local ethics committee.

Patient characteristics are summarized in Table 1. The median age was 71 years (range: 57–87), median IPI was 3 (range: 3–4), median previous lines of treatment was 2 (range: 1–6). No patient had associated myeloid neoplasm on initial staging of bone marrow biopsy and blood count.

**Table 1.** Clinical Characteristics and Patient Follow-up

ID	Age/Sex	Phenotype (IHC)	Mutations (VAF%)	IPI at Diagnosis	Number of Previous Therapy	Previous Auto-HSCT	5-Azacytidine-Venotoclax (No. of Cycles)	Best Response	Allo-HSCT	Relapse/Outcome	Overall Survival^*a*^(months)
**AITL1**	60/M	CD10+PD1+ BCL6+CXCL13–BCL2±EBV–	IDH2 p.Arg172Lys (5.8%) TET2 p.Tyr559Ter (26%)	3	6	1	6	CR	Yes	No/died^*b*^	7.4
**AITL2**	71/M	CD10+PD1+ BCL2+BCL6+ CXCL13+ EBV–	IDH2 p.Arg172Lys (11.7%) RHOA p.Gly17Val (4.4%) TET2 p.Pro1131AsnfTer10 (19.1%)	4	1	0	10	CR	No	No/alive	9.2
**AITL3**	87/F	CD10+PD1+ BCL6+CXCL13+ BCL2+ EBV+	DNMT3A p.Arg882Cys (33.3%) IDH2 p.Arg172Ser (15.6%) RHOA p.Gly17Val (5%) TET2 p.Arg1465Ter (21.2%) TET2 p.Leu1322Gln (11.5%)	4	2	0	12	CR	No	No/alive	11.5
**AITL4**	80/F	CD10+PD1+ BCL6+ CXCL13–BCL2±EBV–	IDH2 p.Arg172Lys (3.4%) RHOA p.Gly17Val (5.5%) TET2p.Glu1106ValfsTer23 (3.6%)	3	2	0	5	PD	No	Yes/died	5
**AITL5**	57/M	CD10+PD1+ BCL6+CXCL13+ BCL2+EBV–	DNMT3A p.Tyr735Thrfs44 (7%)	3	4	0	5	PR^*c*^	No	Yes/alive	8.5

^*a*^The OS reported is from date of initiation of HMA + venetoclax.

^*b*^The patient died on the 34th day after transplantation secondary to veno-occlusive disease.

^*c*^The patient achieved partial response after 3 cycles of 5-Azacytidine+ venotoclax, however the treatment was stopped after cycle 5 because he developed pulmonary aspergillosis and he relapsed 1 month later.

CR = complete response; IHC = immunohistochemistry; HSCT = hematopoietic stem cell transplantation; HMA = hypomethylating agent; IPI = international prognostic indices; OS = overall survival;PD = progression of disease; PR = partial response; VAF = variant allele frequency.

All patients had received CHOP-like therapy and 1 patient had received previous autohematopoietic stem cell transplantation (HSCT). All received a median of 6 cycles (range 5–12 cycles) of 5-azacytidine plus venetoclax. One patient received additional rituximab because he had Epstein-Barr virus replication in the lymph node biopsy with 40% of EBER+ B cells.

The OR and CR rates were 80% and 60%, respectively (Figure 1). One patient underwent allo-HSCT after achieving CR. He died on the 34th day after transplantation secondary to veno-occlusive disease. Two patients are still receiving treatment (Figure 2). Three out of 5 patients are alive. After a median follow-up of 8.5 months (range: 5–11), the median PFS is 7.54 months. The median OS is not reached. The OS at 1 year is 60%.

**Figure 2.**
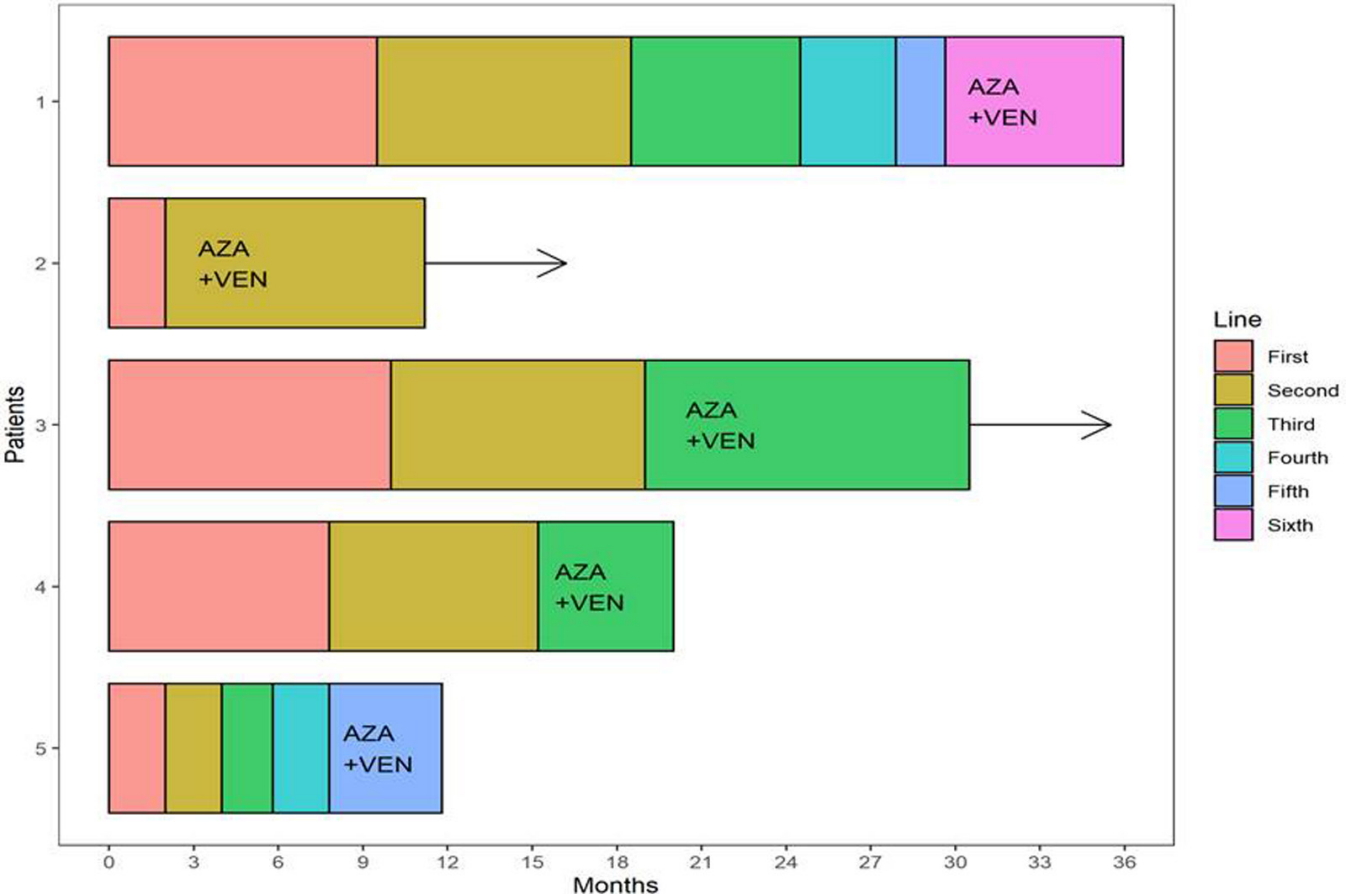
**Swimming plots showing in months the course of treatment for each patient from the diagnosis.** AZA + VEN = azacytidine and venetoclax.

Most of the adverse events were hematological toxic effects. Neutropenia was reported in 5 patients including neutropenia grade 4 in 2 patients and febrile neutropenia in 1 patient.

NGS analyses showed *RHOA G17V* mutations in 3 patients (60%). The epigenetic regulator *TET2* was mutated in 4 patients (80%), and 1 out of 5 patients (20%) had 2 mutations. IDH2 mutations were detected in 4 patients (80%), while DNMT3A were detected in 3 (60%).

Furthermore, the median variant allele frequency (VAF) of *DNMT3A* (20.1%) and *TET2* mutations (20.1%) was higher than that of *RHOA* (5%) and *IDH2* mutations (8.7%). These results are in direct lines with previous reports, suggesting that *DNMT3A* and *TET2* mutations occur earlier and probably in precursor cells.^1,2,12^

The rationale for using epigenetic therapies in AITL is supported by several studies that have shown mutations in epigenetic genes. However, the mechanism of action of HMAs in AITL has not been clarified yet. It is hypothesized that HMA act on regulators of DNA methylation, supported by the overlap with the molecular signature of MDS. Nevertheless, previous studies reported a robust methylation immunophenotype profile in PTCL samples including AITL with the loss of 5-hydroxymethylcytosine in the absence of genetic alterations in the *TET2*, *DNMT3A*, and *IDH2* epigenetic modifiers.^13^ This may explain the response to HMA irrespective of the mutational profile including *TET2* mutational status in previous case reports,^14^ but it remains unanswered as to whether this may confer specific sensitivity to HMAs. Is there a direct effect on neoplastic T cells or other mechanisms?

The BH3-mimetic, venetoclax is able to reinstate the apoptotic potential of tumor cells and therapy resistance induced by overexpression of Bcl-2 or loss of BH3-only protein function.

However, durable response to venetoclax is attenuated by a variety of distinct resistance mechanisms including increased expression of antiapoptotic MCL-1 or BCL-XL leading to maintained cell survival and proliferation. Recent studies showed that the efficacy of venetoclax was improved when combined with agents down regulating MCL1 or BCL-XL such HMAs.^15^

These findings highlight the possible beneficial effects of a 5-azacytidine + venetoclax regimen with acceptable tolerance. However, a longer follow-up is needed. Further trials with ancillary molecular studies are required for a better understanding of this combination.

## ACKNOWLEDGMENTS

The authors appreciate the contributions of the physicians and data manager who made this analysis possible, as well as the contributions made by the patients themselves.

## DISCLOSURES

Outside this work, KL has received research grants from AbbVie, Novartis, Takeda, Roche, Amgen, and Sandoz as well as personal fees from AbbVie, Novartis, Sandoz, Celgene, Jansen, and Amgen. The other authors have no conflicts of interest to disclose.

## References

[1] Lemonnier F Couronné L Parrens M. Recurrent TET2 mutations in peripheral T-cell lymphomas correlate with TFH-like features and adverse clinical parameters. Blood. 2012;120:1466–1469. 22760778 10.1182/blood-2012-02-408542

[2] Cairns RA Iqbal J Lemonnier F. IDH2 mutations are frequent in angioimmunoblastic T-cell lymphoma. Blood. 2012;119:1901–1903. 22215888 10.1182/blood-2011-11-391748 PMC3293643

[3] Laribi K Alani M Truong C. Recent advances in the treatment of peripheral T-cell Lymphoma. Oncologist. 2018;23:1039 1053. 29674443 10.1634/theoncologist.2017-0524 PMC6192612

[4] Bejar R Lord A Stevenson K. TET2 mutations predict response to hypomethylating agents in myelodysplastic syndrome patients. Blood. 2014;124:2705–2712. 25224413 10.1182/blood-2014-06-582809 PMC4208285

[5] Cheminant M Bruneau J Kosmider O. Efficacy of 5-azacytidine in a TET2 mutated angioimmunoblastic T cell lymphoma. Br J Haematol. 2015;168:913–916. 25312805 10.1111/bjh.13170

[6] Lemonnier F Dupuis J Sujobert P. Treatment with 5-azacytidine induces a sustained response in patients with angioimmunoblastic T-cell lymphoma. Blood. 2018;132:2305–2309. 30279227 10.1182/blood-2018-04-840538

[7] Jung JT Kim DH Kwak EK. Clinical role of Bcl-2, Bax, or p53 overexpression in peripheral T-cell lymphomas. Ann Hematol. 2006;85:575–581. 16673127 10.1007/s00277-006-0127-z

[8] D’Aguanno S Del Bufalo D. Inhibition of anti-apoptotic Bcl-2proteins in preclinical and clinical studies: current overview in cancer. Cells. 2020;9:1287. 10.3390/cells9051287 PMC7291206 32455818

[9] Cyrenne BM Lewis JM Weed JG. Synergy of BCL2 and histone deacetylase inhibition against leukemic cells from cutaneous T-cell lymphoma patients. Blood. 2017;130:2073–2083. 28972015 10.1182/blood-2017-06-792150 PMC5680613

[10] King ALO Mirza FN Lewis JM. B-cell lymphoma 2 inhibitor venetoclax treatment of a patient with cutaneous T-cell lymphoma. JAAD Case Rep. 2021;8:89–92. 33537387 10.1016/j.jdcr.2020.12.025 PMC7838714

[11] Cheson BD Pfistner B Juweid ME. Revised response criteria for malignant lymphoma. J Clin Oncol. 2007;25:579–586. 17242396 10.1200/JCO.2006.09.2403

[12] Dobay MP Lemonnier F Missiaglia E. Integrative clinicopathological and molecular analyses of angioimmunoblastic T-cell lymphoma and other nodal lymphomas of follicular helper T-cell origin. Haematologica. 2017;102:e148–e151. 28082343 10.3324/haematol.2016.158428 PMC5395128

[13] Lemonnier F Poullot E Dupuy A. Loss of 5-hydroxymethylcytosine is a frequent event in peripheral T-cell lymphomas. Haematologica. 2018;103:e115–e118. 29242297 10.3324/haematol.2017.167973 PMC5830377

[14] Gregory GP Dickinson M Yannakou CK. Rapid and durable complete remission of refractory AITL with Azacitidine treatment in absence of TET2 mutation or concurrent MDS. HemaSphere. 2019;3:e187. 31723826 10.1097/HS9.0000000000000187 PMC6746031

[15] Yue X Chen Q He J. Combination strategies to overcome resistance to the BCL2 inhibitor venetoclax in hematologic malignancies. Cancer Cell Int. 2020;20:524. 33292251 10.1186/s12935-020-01614-z PMC7597043

